# SIRT1 Deficiency, Specifically in Fibroblasts, Decreases Apoptosis Resistance and Is Associated with Resolution of Lung-Fibrosis

**DOI:** 10.3390/biom10070996

**Published:** 2020-07-02

**Authors:** Raanan Bulvik, Raphael Breuer, Mona Dvir-Ginzberg, Eli Reich, Neville Berkman, Shulamit B. Wallach-Dayan

**Affiliations:** 1 Lung Cellular and Molecular Biology Laboratory, Institute of Pulmonary Medicine, Hadassah—Hebrew University Medical Center, POB 12000, Jerusalem 91120, Israel; rbulvik@gmail.com (R.B.); raffibreuer@gmail.com (R.B.); neville@hadassah.org.il (N.B.); 2 Department of Pathology and Laboratory Medicine, 670 Albany St, 4th Floor, Boston University School of Medicine, Boston, MA 02118, USA; 3 Institute of Dental Sciences, Faculty of Dental Medicine, Hebrew University-Hadassah, POB 12065, Jerusalem 9112102, Israel; monad@ekmd.huji.ac.il (M.D.-G.); reich.eli@gmail.com (E.R.)

**Keywords:** myofibroblasts, SIRT1, cell-death, FLIP, Ku70, IPF-resolution

## Abstract

In contrast to normal regenerating tissue, resistance to Fas- and FasL-positive T cell-induced apoptosis were detected in myofibroblasts from fibrotic-lungs of humans and mice following bleomycin (BLM) exposure. In this study we show, decreased FLIP expression in lung-tissues with resolution of BLM-induced fibrosis and in isolated-lung fibroblasts, with decreased resistance to apoptosis. Using a FLIP-expression vector or a shFLIP-RNA, we further confirmed the critical need for FLIP to regain/lose susceptibility of fibrotic-lung myofibroblast to Fas-induced apoptosis. Our study further show that FLIP is regulated by SIRT1 (Sirtuin 1) deacetylase. Chimeric mice, with SIRT1-deficiency in deacetylase domain (*H355Y*-*Sirt1^y/y^*), specifically in mesenchymal cells, were not only protected from BLM-induced lung fibrosis but, as assessed following Ku70 immunoprecipitation, had also decreased Ku70-deacetylation, decreasedKu70/FLIP complex, and decreased FLIP levels in their lung myofibroblasts. In addition, myofibroblasts isolated from lungs of BLM-treated miR34a-knockout mice, exposed to a miR34a mimic, which we found here to downregulate SIRT1 in the luciferase assay, had a decreased Ku70-deacetylation indicating decrease in SIRT1 activity. Thus, SIRT1 may mediate, miR34a-regulated, persistent FLIP levels by deacetylation of Ku70 in lung myofibroblasts, promoting resistance to cell-death and lung fibrosis.

## 1. Introduction

We have previously demonstrated that CD4^+^ T-cells induce apoptosis in fibrotic lung myofibroblasts [1,2] and that the Flice-like-inhibitory-protein (FLIP) diverts fibrotic-lung myofibroblast Fas-induced apoptosis towards proliferation [3]. Histone deacetylases (HDACs) are involved in stress response and aging [4], and in particular the class III-SIRT1, are associated with fibrotic diseases [5,6,7]. SIRT1 expression is significantly elevated in fibrotic areas of lungs from patients with idiopathic pulmonary fibrosis (IPF) and in the experimental model of bleomycin (BLM)-induced lung fibrosis [8]. It was recently recognized that most deacetylations occur on lysine residues of non-histone targets relevant to cell survival (reviewed by: [9,10]) such as Ku70 [11]. In cancer cells, SIRT1 inhibition increases Ku70-acetylation, which promotes FLIP (FLICE-like inhibitory protein) downregulation [11], and Ku70-deacetylation stabilizes FLIP and prevents cell death [12]. Some studies have linked SIRT1 to apoptosis inhibition via the effects of miR-34a [13,14]. In this study, we show that human IPF-lung myofibroblast resistance from apoptosis is regulated by SIRT1 and is associated with stabilization of FLIP. Concomitantly, lung tissue-regeneration and resolution of fibrosis was detected in the experimental model with SIRT1 deficiency, in mesenchymal cells in chimeric (*H355Y*-*Sirt1^y/y^*) vs. wild type (WT) mice. We further show, based on previous findings, that miR34a, can be a candidate for SIRT1 regulation and regulation of fibroblast viability. Our studies suggest that inhibition of miR34a-mediated SIRT1 activity, specifically on mesenchymal cells, may play a role in the mechanism affecting FLIP stability, and myofibroblast escape from immune surveillance with subsequent accumulation and lung fibrosis.

## 2. Materials and Methods

### 2.1. Human IPF- and Control Lung Myofibroblasts

Anonymized samples were obtained from Dr. Carol Feghali-Bostwick, while at Pittsburgh-University Medical-Center, Pittsburgh, PA, USA [15], under approval of the University of Pittsburgh Institutional Board. LL 97A (AlMy) (ATCC^®^ CCL-191™)-IPF-lung, and LL 24 (ATCC^®^ CCL-151™)-normal lung fibroblast cell lines were also used.

### 2.2. Mice

Male, 11–12 wk C57BL/6 WT, (Harlan, Indianapolis, IN, USA) or Sirt1^tm2.1Mcby^ (Sirt1^y/y^, RBRC05324, RIKEN Bio Resource Center, Tsukuba, Japan) and deleted miR-34a pre-miRNA, kindly provided by Prof. Yinon Ben-Neria, Hebrew University, Jerusalem, Israel, approved by the Institutional Board of the Hadassah-Hebrew University Medical Center (Research Permission MD-15-14590-5, issued on 16.12.2015), were used. Four to six mice were used for each time point of each experiment.

### 2.3. Chimeric Mice

Sirt1^tm2.1Mcby^ chimeric mice with SIRT1-deficient mesenchymal cells, and WT hematopoietic cells, were generated with the same procedure as described by us previously for the generation of chimeric gld-mice [16]. Thirty days later, the mice that were tested for chimerism [16] introduced exposed to bleomycin or saline and fibrosis assessed.

### 2.4. Oropharyngeal Aspiration (OA) Experimental Model

OA of bleomycin (OA-BLM) was performed and fibrosis assessed by; the semi-quantitative morphological index, newly formed collagen deposition by sircoll assay, or staining in lung tissue sections, as previously detailed [2,16].

### 2.5. Mouse Lung Myofibroblasts

Mouse lung myofibroblasts were isolated from the lungs of mice, at different time points of BLM treatment, as described by us in detail elsewhere [2,16].

### 2.6. Cell Death and Apoptosis

Apoptosis was determined by Annexin V affinity labeling, or caspase-3 cleavage in Western blot (WB) and cell-viability by trypan-blue exclusion. All as we described previously [2,16,17].

### 2.7. Immunohistochemistry (IHC) Staining of Lung Tissue Sections

IHC staining was performed in lung tissue sections of mice, at different time points of BLM treatment, or in lung tissue sections from IPF patients, as we previously described [2,16] using anti-FLIP monoclonal antibody (mAb) and SIRT1 mAb.

### 2.8. Semiquantitative Morphological Index (SMI) Grading

Semiquantitative morphological index was assigned on evaluation of trichrome-stained lung sections without knowledge of treatment groups, with the grades as follows: 0, normal lung; 1, minimal areas of inflammation, epithelial hyperplasia and fibrosis, usually limited to subpleural foci; 2, more frequent lesions; 3, section exhibits lung lesions which are not limited to subpleural foci; 4, extensive lesions in most of the section; and 5, majority of section affected by inflammation and fibrosis [18].

### 2.9. FLIP Protein in Lung Myofibroblasts

Standard WB, cell lysis, immunoprecipitation and flow cytometry were performed in isolated lung fibroblasts, as we described previously using anti-FLIP mAb [3,19].

### 2.10. FLIP up/downregulation

FLIP-long cDNA expression vector (2 μg), and control (pcDNA3.1 vector alone), kindly supplied by Dr. Dan Longley (Queen’s University of Belfast, Ireland, UK), were applied [20] to 5 × 10^5^ FLIP^low^ fibroblasts, using transfection kit (Cat. No. MPK-1096; Thermo Fisher Scientific, Waltham, MA USA) and a microporator, with 12 µL of “Solution R” (transfection kit). shRNA was designed, from the GeneBank (NM009805), with 5′ overhangs on each side for direct ligation into the vector (Integrated DNA Technologies, Coralville, IA, USA). Top strand: 5′CGCGTCCCCGAATAGACTTGAACACAAATTCAAGAGATTTGTGTTCAAGTCTATTCTTTTTGGAAAT3′.

Bottom strand: 5′CGATTTCCAAAAAGAATAGACTTGAACACAAATCTCTTGAATTTGTGTTCAAGTCTATTCGGGGA3′

The GFP+pLVTHM lentiviral vector was generously provided by Prof. Darrell Kotton (BU-Boston, MA, USA). Cell transductions were performed for 24 h with the vector (200 MOI) with 8 µg of polybrene (Sigma Aldrich) [21].

### 2.11. Mimic-miR34a Transfection

Mimic-miR34a transfection was by using miRIDIAN microRNA hsa-miR-34a-3p mimic (5′-UGGCAGUGUCUUAGCUGGUUGU-3′) or negative control-1 (Dharmacon, Tamar, Israel), in a concentration of 50 nM for 72 h, using Trans-IT-X2 transfection reagent (Mirus, Zotal, Israel).

### 2.12. RNA Analysis and Quantitative PCR (qPCR)

Total fibroblast’s RNA extraction by RNeasy Kit (Qiagen, Hilden, Germany), and reverse-transcription using M-MLV-RT (Quanta, Biological Industries, Beit HaEmek, Israel). Real-time PCR (Rotergene, Qiagen), with SYBR GREEN (Agentec, Applied Biosystems, Warrington, UK), using suitable primers (Table 1).

### 2.13. Luciferase Assay

293T cells were transfected with 200 ng pmirGLO-hSIRT1, 3′ UTRs of human SIRT-1, or a pmirGLO-control (Kiga Katora [14]), and 50 nM mimic-miR-34a or negative-control (Dharmacon, Lafayette, CO, USA), using 0.5 mL Trans-IT×2 transfection reagent (Mirus-Bio, Madison, WI, USA). Firefly and Renilla Luciferase were measured with the Dual-Luciferase assay (Promega, Madison, WI, USA) 72 h after, by Mitras laminator (Berthold, Bad Wildbad, Germany).

### 2.14. Data Analysis and Statistics

The Kruskall–Wallis to compare variables at different times or treatments. Mann–Whitney test with the Bonferroni correction, to test significance. Two-way ANOVA for time/treatment interactions. All data is presented as mean ± standard error.

## 3. Results

### 3.1. Lungs of BLM-Treated Mice Resolving Fibrosis Decrease FLIP and Myofibroblasts Regain Susceptibility to T Cell-Induced Apoptosis

**Figure 1:**
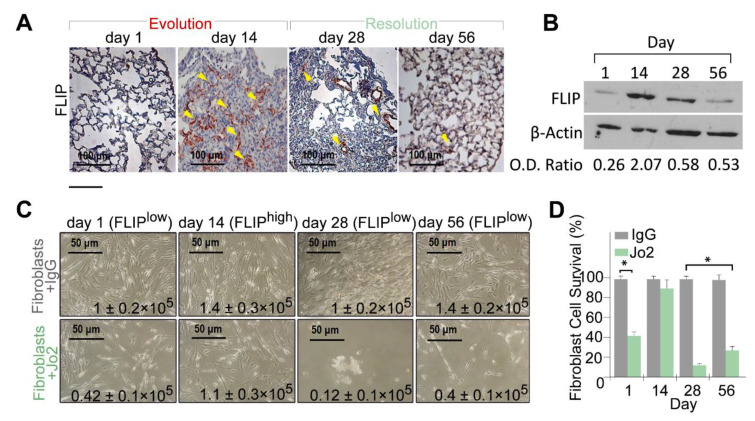
Lung fibrosis resolution in mice correlates with loss of Flice-like-inhibitory-protein (FLIP) and of myofibroblast viability. (**A**) FLIP (arrows) in bleomycin (BLM)-treated murine lung tissue, before fibrosis (day 1), with fibrosis (day 14) and at resolution (day 28, 56). Representative of 15 fields (×20) in each mouse. *n* = 4 for each time point. (**B**) Representative western blot of FLIP protein expression in 3 × 10^5^ myofibroblasts isolated from lungs of mice at days 1, 14, 28, and 56 post BLM. (**C**) Light microscopy images with trypan blue exclusion (inserted numbers), and (**D**) graphical presentation of cell viability (%), in control-IgG vs. Jo2 (20 µg, 48 h) anti-Fas mAb-treated fibroblasts from days 1, 14, 28, and 56 post BLM. * *p* = 0.021.

We previously showed increased FLIP levels during fibrosis evolution [3,22] in lungs, and isolated myofibroblasts, from C57BL/6 mice 14 days following BLM exposure. Fibroblasts from lungs with fibrosis with high FLIP levels were defined as FLIP^high^ fibroblasts. Here we continued to evaluate FLIP kinetics at resolution of BLM injury [1], and show, in vivo in lung-tissue sections, that FLIP gradually returns to baseline during resolution of lung fibrosis (Figure 1A; FLIP and hematoxylin counter staining) at days 28 and 56 vs. day 14. This was further confirmed in western blot, in cell-lysates from isolated-lung fibroblasts, showing an optical density (OD) decrease from 2.07 at day 14, to approximately 0.5, at days 28 and 56 (Figure 1B). These fibroblasts were defined as FLIP^low^ fibroblasts. In parallel experiments, we cultured fibroblasts isolated from days 1, 14, 28, and 56, and exposed them to agonist anti-Fas antibody (Jo2) vs. control IgG. Myofibroblasts were susceptible to cell-death at day 1, resistant at day 14, and gradually regain cell death at day 28 to day 56. Their number, at day 28 and 56 decreased from a peak of 1–1.4 × 10^5^ (fibroblasts + IgG) to only 0.12–0.42 × 10^5^ (fibroblasts + Jo2) while the number of fibrotic-lung myofibroblasts, at day 14, had a non-significant change following anti-Fas exposure (i.e., 1.4–1.1 × 10^5^), (Figure 1C). Graphical presentation of myofibroblasts cell survival, with statistical analysis, demonstrate that fibroblasts isolated from BLM-treated lungs at day 14 (FLIP^high^) show no significant difference in cell viability between anti-Fas and control IgG (Figure 1D, Day 14). However, those isolated from lungs during resolution (FLIP^low^), are susceptible to cell death, and their viability decreases by almost 80% (Figure 1D, day 28 and 56).

### 3.2. FLIP Downregulation is Critical to Regain Fibrotic-Lung Myofibroblast Predisposition to Fas-Induced Apoptosis and for Attenuation of Lung Fibrosis

**Figure 2:**
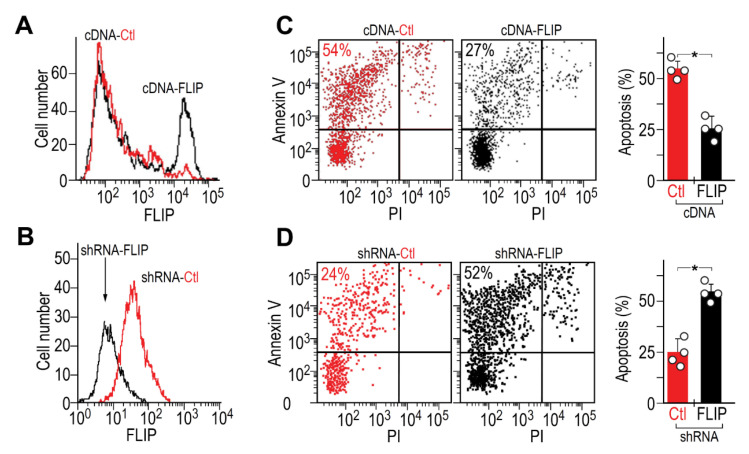
Critical role of FLIP in myofibroblast resistance to apoptosis and BLM-induced lung fibrosis. Flow cytometry of FLIP expression in myofibroblasts (αSMA^+^), isolated from (**A**) lungs resolving fibrosis (cDNA-Ctl, low FLIP), compared to those transfected with FLIP cDNA expression vector (cDNA-FLIP) and from (**B**), fibrotic-lungs (shRNA-Ctl, high FLIP), transduced with shRNA-FLIP-GFP^+^ lentiviral vector (shRNA-FLIP). (**C**,**D**) Flow cytometry of Annexin V staining (left pair of panels) with graphical presentation (right panel), of the percentage of apoptotic transfected/transduced vs. control myofibroblasts, following Fas exposure. *n* = 4 * *p* < 0.05.

We overexpressed FLIP, in initially FLIP^low^ myofibroblasts from lungs resolving fibrosis (day 56), and downregulated FLIP, via FLIP shRNA GFP^+^ lentiviral vector, in initially FLIP^high^ myofibroblasts from fibrotic lungs (day 14). We determined FLIP changes by flow cytometry (Figure 2A,B, respectively). Transfected/transduced fibroblasts, who kept FLIP levels for up to three passages, were exposed to anti-Fas Jo2 mAb and analyzed for apoptosis by Annexin V staining flow cytometry (Figure 2C,D, respectively). Dot-plot pairs of control and transfected, on the left with graphical presentation, on the right.

FLIP overexpression, in originally FLIP^low^ fibroblasts (Figure 2A, cDNA-FLIP, black histogram vs. red), increased in half of the cells from a mean fluorescence intensity (MFI) of 10^2^ to 10^4^. Concomitantly, Annexin V staining decreased from 54% to 27% (Figure 2C, dot plot pair and graphical presentation of cDNA-FLIP compared to cDNA-Ctl). In contrast, FLIP downregulation, in originally FLIP^high^ fibroblasts, by shRNA (Figure 2B, shRNA-FLIP, black histogram vs. control in red), decreased FLIP from 100 to only 5 mean fluorescence intensity and more than doubled the number of apoptotic cells (Figure 2D, dot plot pair and graphical presentation of shRNA-FLIP compared to shRNA-Ctl). These data corroborate the critical need for FLIP downregulation in lung myofibroblasts to regain susceptibility to Fas-induced apoptosis.

### 3.3. SIRT1, and Ku70-Deacetylations, are Increased in Human IPF- and in BLM-Lung Myofibroblasts. Concomitantly, BLM-Treated Chimeric Mice, with Specific SIRT1-Deficiency, in Mesenchymal Cells, Increase Acetylated-Ku70, Decrease FLIP and Show Less Fibrosis

**Figure 3:**
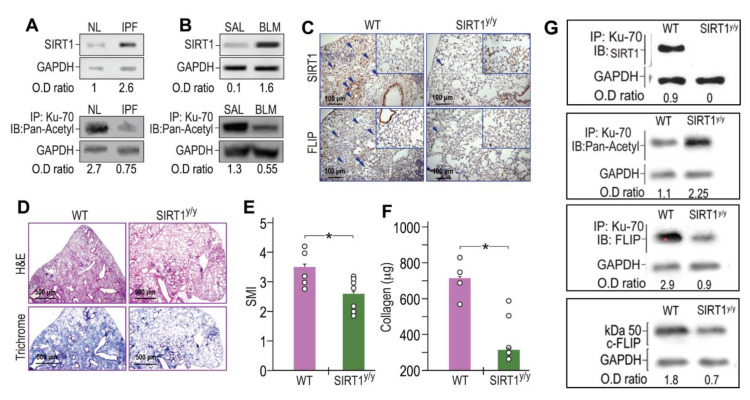
SIRT1 and Ku70-deacetylations increased in human IPF- and in BLM-lung myofibroblasts. Concomitantly, BLM-treated chimeric mice, with SIRT1-deficiency, specifically in myofibroblasts, downregulate FLIP, and show less fibrosis. Fibroblasts were isolated from lungs of patients with IPF- vs. normal subjects, and from BLM- or saline (SAL) - treated mice fourteen days after treatment. (**A**,**B**, upper panels) SIRT1 immunoblot (IB) and (**A**,**B**, lower panels) Ku70 immunoprecipitation (IP) with subsequent pan-acetyl IB (*n* = 4). (**C**,**D**) IHC in lung tissue sections of chimeric, *SIRT1^y/y^* vs. WT, mice, 14 days post BLM (×20 and ×40 inserts, arrows indicate representative stained areas). SIRT1 (**C**, upper panels), FLIP (**C**, lower panels), H&E (**D**, upper panels), and Trichrome (**D**, lower panels). (**E**) Graphical presentation of semi-quantitative morphology index (SMI) of H&E stained sections, and (**F**) collagen Sircol assay. (**G**) Fibroblasts isolated from lungs of WT or SIRT1^y/y^ mice; Ku70 IP with subsequent IB with mAbs for SIRT1, Pan-Acetyl, FLIP, and total lysate IB for FLIP. Representative of two experiments. *n* = 4–5. * *p* < 0.05.

Fibrotic-lung myofibroblasts both from humans with IPF (Figure 3A) and BLM-treated mice lungs (Figure 3B) have increased SIRT1 (Figure 3A,B, upper panels) with reduced Ku70 acetylation (Figure 3A,B, lower panels) when compared to their normal counterparts (“IPF vs. NL” or “BLM vs. SAL”). We then studied the mechanism whereby the increased deacetylation of Ku70, detected in fibrotic-lung fibroblasts, is dependent on SIRT1 enzymatic activity. Although FLIP can be regulated at the transcription level by histone deacetylase HDAC3, 6, and 8 [23], like previous studies that suggest regulation of FLIP at a post-translational level [11,12] we aimed to assess, whether SIRT1 leads to the increments in Ku70-FLIP complex and FLIP accumulation. To this end, we determined FLIP levels, its complex with SIRT1 or Ku70, Ku70 deacetylation, and fibrosis evolution following bleomycin injury, in mice in which mesenchymal cells have a specific SIRT1 mutation on lysine-substrate binding site (*SIRT1^y/y^* C57BL/6 “chimeric” mice). BLM-treated *SIRT1^y/y^* chimeric mice, with normal hematopoietic cells but with mesenchymal cells that bear an inactive SIRT1, were used to determine in vivo effects of SIRT1’s deficiency in mesenchymal cells (e.g., fibroblasts), following BLM-exposure. Fourteen days after BLM-treatment, *SIRT1^y/y^* chimeric-mice had significantly lower FLIP levels compared to WT control chimeric-mice as assessed by IHC in lung-tissue sections (Figure 3C, lower panels). We have repeatedly shown (in circumstances leading to SIRT1 absence in particular [24]), both immunohistochemical staining of the trichrome or that of the α-smooth muscle actin, indicate on increments or decrements in myofibroblast population and activity and can interchange. In this study we determined the absence of fibrosis in BLM-treated SIRT1^y/y^ mice and found a pronounced fibrosis reduction, determined by hematoxylin-eosin and collagen-trichrome staining in lung tissue sections (Figure 3D, upper and lower panels, respectively). Moreover, BLM-treated *SIRT1^y/y^* slightly differ from that of saline-treated *SIRT1^y/y^* mice which did not differ from saline-treated WT mice and showed no differences in fibrosis markers, or FLIP (Figure 3C,D, inserts in lower and upper panels). A semi quantitative morphological index grading of H&E-stained sections, revealed a 30% reduction in *SIRT1^y/y^*-mouse lung fibrosis compared to WT (Figure 3E) but soluble collagen content was decreased by over 80% in Sircol assay **(**Figure 3F), and lymphocytes fell from an average of 26% to only 4% in bronchoalveolar analysis (not shown). Fibroblasts were then isolated from lungs of WT or *SIRT1^y/y^* mice to assess the direct binding of SIRT1 to Ku70 substrate and its absence in SIRT1-deficiency. Ku70 immunoprecipitation (IP) with subsequent immunoblots (IB) with mAbs for SIRT1, Pan-Acetyl, FLIP, and IB of FLIP, clearly show that Ku70 does not bind to SIRT1 active site. Ku70 acetylation was increased from 1.1 to 2.25 (OD ratio to glyceraldehyde-3-phosphate dehydrogenase, or GAPDH), its binding to FLIP decreased from an OD ratio of 2.9 to 0.9 and FLIP levels subsequently decreased from1.8 to 0.7 (Figure 3G). Thus, poor SIRT1 activity, specifically in mesenchymal cells (e.g., myofibroblasts, epithelial cells and those under epithelial-mesenchymal transition), plays a role in FLIP destabilization by decreasing Ku70 acetylation, may be critical for fibrosis resolution.

### 3.4. Human IPF-Lung Myofibroblasts Decrease SIRT1 to Normal Levels by a miR-34a Mimic, and Similar to miR-34a Mimic-Treated miR34KO-Murine Lung Myofibroblasts, Decrease Ku70-Deacetylation, Ku70-FLIP Complex, and FLIP

**Figure 4:**
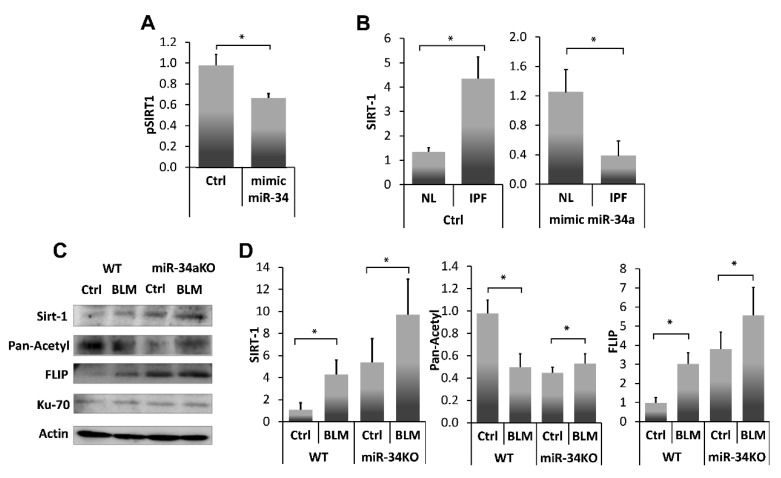
SIRT1 levels, and activity, in fibrotic-lung myofibroblasts, can be regulated by a miR-34a mimic. (**A**) Luciferase reporter assay using a Mitras laminator. PmirGLO-hSIRT1 (pSIRT1)-transfected 293-T cells treated with control or mimic-miR34a (200 nM, 72 h) (Ctrl vs. mimic miR34, respectively). (**B**) SIRT1 expression (qPCR), in cells from fibrotic/IPF vs. normal-lung (NL) myofibroblast cell lines, following miR-34a control (200 nM, 72 h) exposure (**B**, Left panel, saline (Ctrl) in NL and IPF), and after exposure (**B**, Right panel, mimic miR34a in NL and IPF). Fibroblasts were isolated from lungs of Ctrl-or BLM-treated WT and miR34a knockout mice. (**C**) Ku70 IP with subsequent IB and, (**D**) graphical presentation of optical density (OD) ratios to β-actin (the product of a housekeeping gene) for SIRT-1, pan-acetyl, and FLIP. Representative from four independent experiments, *n* = 4–6. * *p* < 0.01–0.05.

miR-34a is known to repress SIRT1 affecting susceptibility to apoptosis [13], particularly in fibrotic lung fibroblasts [25]. In this study (Figure 4A), we further verified, SIRT1 regulation by miR-34a, using a miR34a mimic, in lung myofibroblasts isolated from lungs of mice on day 14 post BLM instillation. Initially, we confirmed SIRT1 regulation with our miR-34a mimic in a luciferase reporter assay following transfection of 293-T cells with SIRT1 3′UTR. Luciferase activity was decreased by 30% compared to negative controls when SIRT1-transfected cells were treated with the mimic-miR34a (200 nM, 72 h), (Figure 4A, pSIRT1+mimic miR34a vs. pSIRT1+saline (Ctrl), respectively). Thereafter, cells from a human lung myofibroblast IPF cell line (i.e., CCL-191) that express high SIRT1 (Figure 4B, IPF vs. normal-lung (NL) left panel) were treated with the mimic-miR34a. SIRT1 mRNA was quantified by qPCR and compared with negative controls (Figure 4B, right panel). miR-34a overexpression in human fibrotic myofibroblasts was associated with a three-fold decrease in SIRT1 levels compared to negative control (B, left panel, Ctrl in NL and IPF), and after exposure (Figure 4B, Right panel, mimic miR34a in NL and IPF).

To further assess miR34a regulation on SIRT1 and subsequent interactions of SIRT1 with Ku70, and Ku70-mediated stabilization of FLIP, we determined miR-34a absence effects on the direct binding of SIRT1 to Ku70-SIRT1, and SIRT1deacetylation activity on Ku70, and Ku70 binding to FLIP, in lung fibroblasts from bleomycin-, compared to saline- treated WT and miR-34KO mice. Fibroblasts were isolated from lungs of Ctrl- or BLM-treated WT and miR34a knockout mice 14 days post-treatment. (Figure 4C). Direct binding of Ku70 to SIRT1, FLIP and its acetylation were determined in all cell-lysates following Ku70 immunoprecipitation with subsequent immunoblots and, using specific mAbs to SIRT1 or FLIP or pan-acetyl. A graphical presentation of OD ratios to actin for; SIRT-1, pan-acetyl, and FLIP follows (Figure 4D).

In Ctrl- vs. BLM-treated WT mice, low levels of SIRT1 binding to Ku70, correlated with increased Ku70 acetylation, and with low levels of Ku70-FLIP complex. In contrast, in miR-34KO mice (Ctrl- or BLM-treated), SIRT1 binding to Ku70 increased, Ku70 acetylation decreased, and FLIP-Ku70 complex, increased. In all groups, Ku70 expression, per se, remained constant (Figure 4C,D, I.B Ku70). These results suggest that SIRT1 deacetylation activity on Ku70 is affected by miR-34a which further influence FLIP binding to Ku70.

## 4. Discussion

As we have previously shown, lymphocytes are capable of inducing apoptosis in myofibroblasts during resolution of lung fibrosis, thus acting as an anti-fibrotic defense mechanism [1]; and FLIP is capable of suppressing this cell death, allowing fibroblast to escape the immune response and to proliferate, thus promoting fibrosis [3].

In the current study, which focuses on mechanisms of fibrosis resolution, FLIP expression is not only decreased at resolution phase (Figure 1), but it is critical (Figure 2) in lungs of BLM-treated mice resolving fibrosis, where myofibroblasts regain susceptibility to cell death (Figure 1) and apoptosis (Figure 2). Increasing FLIP expression in fibrotic-lung fibroblasts is based on a mechanism of SIRT1-mediated Ku70-deacetylation, which leads to Ku70/FLIP complex, FLIP stabilization, and fibrosis evolution (Figure 3). Resolution of fibrosis is gained in chimeric-mice with SIRT1-deficient mesenchymal cells. Moreover, the amounts of myofibroblasts isolated from lungs of BLM-treated SIRT1-deficient mice vs. wild-type mice were almost negligible but those that were cultured in vitro showed a markedly-decreased binding of the SIRT1 to its substrate Ku70, which had increased acetylations, and decreased binding to FLIP (Figure 3G). We have recently showed that miR34a is one of the epigenetic factors regulating FLIP possibly mediated by SIRT1 [24]. In this study, a miR34a mimic decreased SIRT1 activity and levels (Figure 4A,B, respectively). Moreover, miR-34 knockout mice lung fibroblasts increase Ku70 immunoprecipitation with SIRT1, decreased its immunoprecipitation pan-acetyl mAb indicating decreased Ku70 acetylation, on par of those of WT animals with BLM-induced fibrosis (Figure 4C,D-graphical presentation). The acetylation levels of Ku-70 in fibrotic lung of mir-34 KO animals thus remain almost stable in comparison to saline-treated control. This is explained by the decreased SIRT1 deacetylation activity concomitant to recently published data demonstrating in the experiments in vitro and in vivo, that miR-34a expression is inversely correlated with FLIP levels in lung myofibroblasts of animals with bleomycin-induced pulmonary fibrosis, and miR-34a-mediated downregulation of FLIP positively affects fibrosis outcome [24].

SIRT1 is a multifunctional protein shown to be involved in fibrosis and aging of various organs [26,27], with particularly contradictory results in BLM-induced lung fibrosis [8,28,29,30]. Differences between findings may result from assessment at different time points of fibrosis following injury and/or of assessment of fibrosis following changing SIRT1 activity vs. changes in SIRT1 expression. In our case, we found increased SIRT1 activity in fibrotic-lung myofibroblasts from IPF patients and BLM-treated mice. Inhibition of SIRT1 deacetylation activity, by a specific mutation on SIRT1 as with *SIRT^y/y^* chimeric mice, was associated with reduced FLIP expression, increased apoptosis cascades, and decreased survival pathways in IPF-lung fibroblasts, with attenuation of experimental fibrosis in mouse lungs. Ku70 acetylation was considerably augmented in the *SIRT^y/y^* mice; supporting the finding that SIRT1 deacetylation on a non-histone Ku70 can stabilize FLIP [12]. Moreover, changes in Ku70 acetylation enable p53 activity [31] and play important roles in blocking Bax-mediated apoptosis [32].

Besides having a negative feedback on itself [33], as detected in *SIRT^y/y^* mice (see Figure 3), SIRT1 is a key factor in the regulation of several apoptosis cascades and survival signaling pathways, particularly by repression of p53 [34]. These findings are supported by others, who have shown that histone modifications on Fas facilitate apoptosis in fibrotic-lung myofibroblasts [35]. In addition, Chua et al. [36] reported that murine embryonic fibroblasts lacking SIRT1 are resistant to senescence in the presence of chronic sublethal oxidative stress. Concomitantly, remodeling and deregulated wound healing with fibrosis, was related with age-associated tissue abnormalities or cellular senescence (for a review see [37]). Sirtuins have been implicated in aging and age-related disease, and SIRT1 function has been examined in the context of cellular senescence (reviewed by Longo et al. [38]). Thus, the current study may shed light on SIRT1 influence in the evolution of lung fibrosis with age.

IPF is thought to be an age-related disease, mostly because a large majority of IPF patients are aged 50 years and older [39,40]. Therefore, the possibility that age per se plays a confounding role in influencing resistance to apoptosis, as is the case in aberrant senescent cells [41], cannot be excluded. Accordingly, the resolution of fibrosis detected in the BLM experimental model should be assessed in aged-mice, where responses are known to more accurately resemble that of human-IPF, since they do not resolve fibrosis [42].

## 5. Conclusions

**Figure 5:**
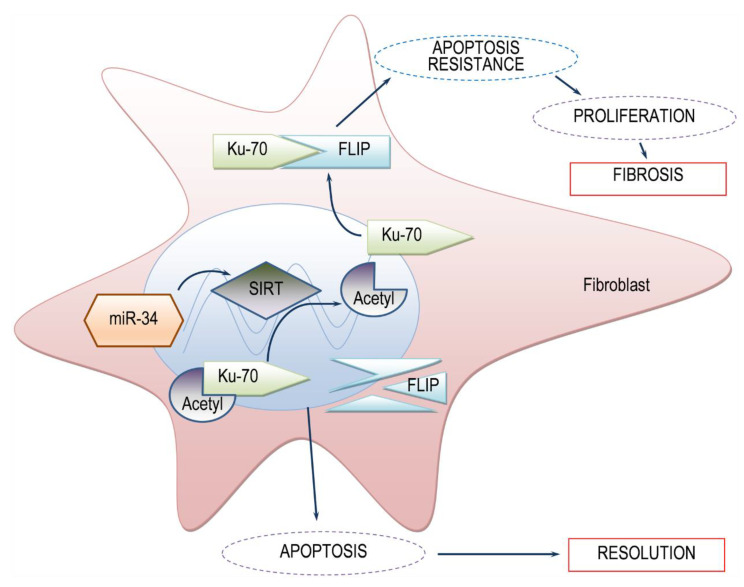
Simplified scheme of the proposed model SIRT1-mediated FLIP regulation during lung fibrosis. In the presence of SIRT1, low levels of acetylated Ku70 are detected. Deacetylated-Ku70 form complexes with FLIP, stabilizing FLIP. Increased FLIP, increases fibroblast resistance to apoptosis and fibrosis occurs. On the other hand, when SIRT1 is absent, Ku70 is in an acetylated state, cannot form complexes with FLIP, FLIP is destabilized, leading to fibroblast apoptosis and subsequent fibrosis resolution. miR34a mimic, may be a candidate for SIRT1 regulation.

Histone deacetylases are known regulatory pathways of Ku70 deacetylation [12]. In our context, transfection of human fibrotic lung myofibroblasts with mimic miR-34a resulted in decreased SIRT1 expression and activity (e.g., decreased Ku70 deacetylation), and decreased FLIP levels (Figure 4). This indicates that high levels of SIRT1 in myofibroblasts of BLM-treated mice can lead to high levels of FLIP, enabling myofibroblast resistance to immune response and advancing fibrosis (See schematic summary in Figure 5).

## Figures and Tables

**Table 1:** Primers for qPCR.

Gene Species	Primer: Forward	Primer: Reverse
*Murine/human U6 snRNA*	GACTATCATATGCTTACCGT	GCGAGCACAGAATTAATACGAC
*Human HPRT*	TGACACTGGCAAAACAATGCA	GGTCCTTTTCACCAGCAAGCT
*Murine HPRT*	GTTAAGCAGTACAGCCCCAAA	GGGCATATCCAACAACAAACTT
*Human SIRT-1*	TCGCAACTATACCCAGAACATAGACA	TGTCATGGTTCCTTTGCAACAG
*Murine SIRT-1*	GCAACAGCATCTTGCCTGAT	GTGCTACTGGTCTCACTT
